# New Medical Journal

**Published:** 1869-06

**Authors:** 


New Medical Journal—*The Journal of the Gynæcological Society of Boston.*—This new Monthly Medical Journal is to contain the official publications of the Gynæcological Society, the first number of which will be issued July 1st. The Journal will be under the editorlal management of the officers of the Society, Drs. Winslow Lewis, H. R. Storer and Geo. H. Bixby. Each number will consist of not less than sixty-four pages, octavo, printed in large type, on fine paper, and it will be furnished to subscribers at three dollars a year, payable in advance.

